# Genomic approaches to trace the history of human brain evolution with an emerging opportunity for transposon profiling of ancient humans

**DOI:** 10.1186/s13100-021-00250-2

**Published:** 2021-10-18

**Authors:** Yilan Wang, Boxun Zhao, Jaejoon Choi, Eunjung Alice Lee

**Affiliations:** 1 Division of Genetics and Genomics, Boston Children’s Hospital and Harvard Medical School, Boston, MA USA; 2 The Broad Institute of Harvard and MIT, Cambridge, MA USA; 3 Program in Biological and Biomedical Sciences, Harvard Medical School, Boston, MA USA; 4 Manton Center for Orphan Disease Research, Boston Children’s Hospital, Boston, MA USA; 5 Department of Genetics, Harvard Medical School, Boston, MA USA

**Keywords:** Transposable elements, Human brain evolution, Ancient human genomes

## Abstract

Transposable elements (TEs) significantly contribute to shaping the diversity of the human genome, and lines of evidence suggest TEs as one of driving forces of human brain evolution. Existing computational approaches, including cross-species comparative genomics and population genetic modeling, can be adapted for the study of the role of TEs in evolution. In particular, diverse ancient and archaic human genome sequences are increasingly available, allowing reconstruction of past human migration events and holding the promise of identifying and tracking TEs among other evolutionarily important genetic variants at an unprecedented spatiotemporal resolution. However, highly degraded short DNA templates and other unique challenges presented by ancient human DNA call for major changes in current experimental and computational procedures to enable the identification of evolutionarily important TEs. Ancient human genomes are valuable resources for investigating TEs in the evolutionary context, and efforts to explore ancient human genomes will potentially provide a novel perspective on the genetic mechanism of human brain evolution and inspire a variety of technological and methodological advances. In this review, we summarize computational and experimental approaches that can be adapted to identify and validate evolutionarily important TEs, especially for human brain evolution. We also highlight strategies that leverage ancient genomic data and discuss unique challenges in ancient transposon genomics.

**Supplementary Information:**

The online version contains supplementary material available at 10.1186/s13100-021-00250-2.

## Introduction

The human brain is widely regarded as the substrate for a multitude of human-specific activities ranging from building complicated tools and using elaborate and abstract language to producing art, science, and distinct cultures [1, 2]. Humans who lived up to hundreds of thousands of years ago and exhibited anatomic features consistent with contemporary humans are referred to as anatomically modern humans (AMHs) in this review. In contrast, archaic humans—other extinct *Homo* species such as the Neanderthals and Denisovans—shared ancestry with AMHs but had a drastically different skeletal shape and anatomic features from AMHs [3]. Compared to closely related primate relatives, AMHs have evolved to possess distinct brain-related anatomic features including the larger neocortex and other brain structures thought to advance processing and storage of information [4]. They also show delayed prenatal and prolonged postnatal brain and neural development that allows for a larger brain and more flexibility for environment-based learning [5–7].

**Fig. 1:**
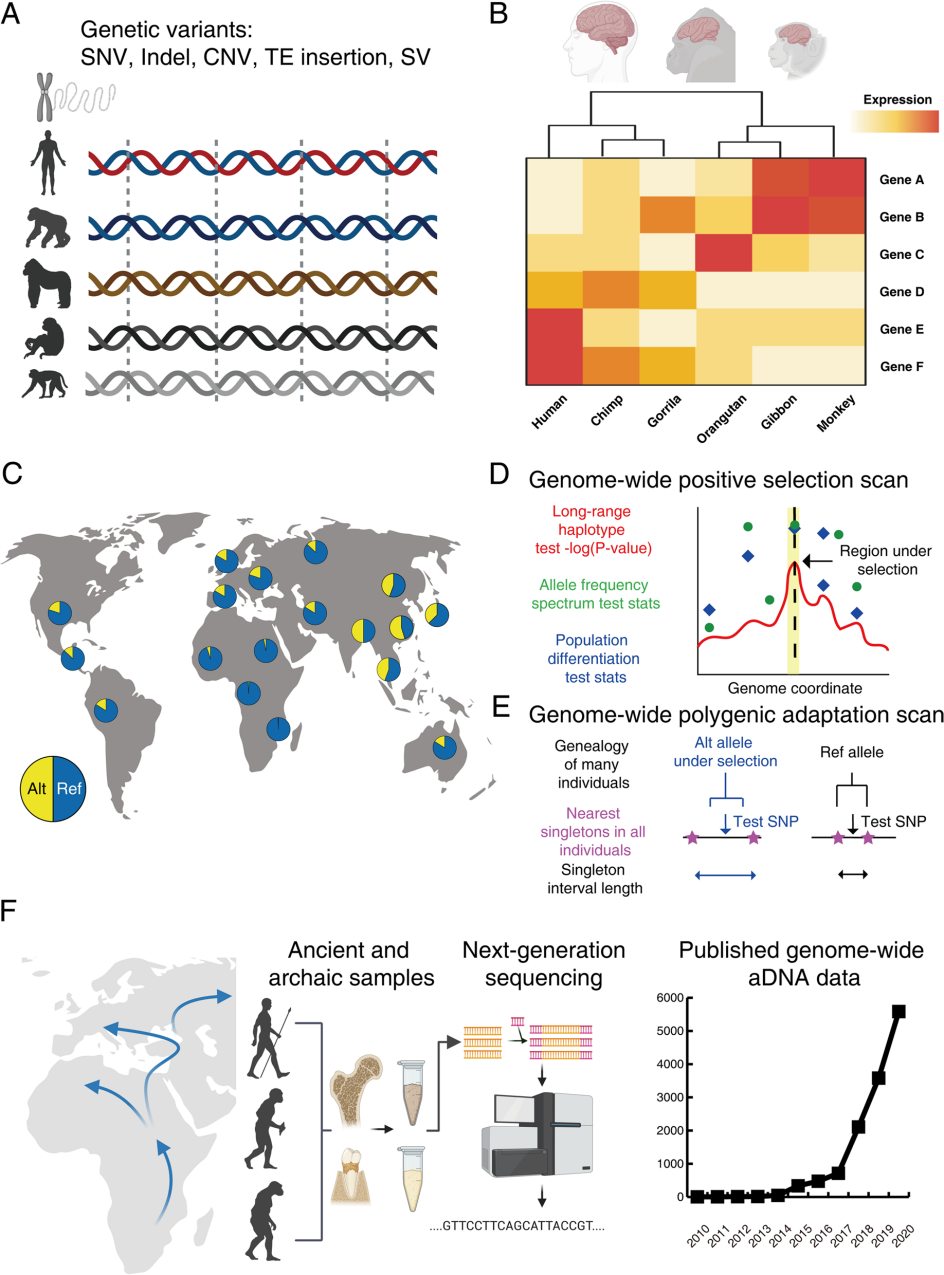
Computational approaches to study the genetic basis of human brain evolution. (**A**) Comparative studies of human and closely related non-human primates (NHPs). Cross-species genomic comparison reveals various types of genetic changes ranging from single nucleotide variants (SNVs) to megabase-scale copy number and structural variants (CNVs and SVs) in orthologous genes and non-coding regions. (**B**) Cross-species transcriptomic analysis identifies spatial and temporal changes in gene expression and RNA splicing during brain development and evolution. (**C**) Population genetic analysis of diverse modern human populations. Differences in allele frequencies of a polymorphic variant (yellow in the pie charts) across different populations may indicate natural selection. (**D**) Genome-wide positive selection scans, including the long-range haplotype test, the allele frequency spectrum test, and the population differentiation test predict genomic regions under positive selection. (**E**) The Singleton-Density-Score method identifies genomic regions under polygenic adaptation by detecting alternative alleles that show unusually short terminal branch lengths due to long intervals between singleton test SNPs in the genealogy tree, compared to the lengths of the corresponding reference alleles. (**F**) Leveraging ancient human genomes to identify genetic variants with allele frequency changes over time. The number of ancient/archaic human samples (WGS and SNP arrays) are shown according to publication years

The scientific community has long sought to understand the evolutionary processes that shaped the unique human brain, with little insight to date [1, 8, 9]. Overall, there are three computational approaches to the study of human brain evolution (Fig. 1). First, studies identify unique genetic/transcriptional/epigenetic changes in humans compared to closely related non-human primates (NHPs). Second, population-genetic studies identify variants under selection by examining genetic variations in diverse modern humans. Third, time-series analysis of ancient and archaic human genomes traces human’s evolutionary trajectory.

Previous studies, however, failed to find strong signals to explain human behavioral changes during the Neolithic period [10]. This might be due to the presence of different types of variants like transposable elements (TEs) that are not in strong linkage disequilibrium (LD) with surveyed single-nucleotide polymorphisms (SNPs) or copy number variants (CNVs) [11, 12], or to combinations of common genetic factors of low effect size that may contribute to brain evolution according to the polygenic hypothesis. This review summarizes recently published human brain evolution-related studies and different statistical and experimental methods conducive to investigating TEs in the context of human brain evolution, suggesting knowledge gaps, technical challenges, potential solutions, and new perspectives. In particular, we highlight the potential in using emerging sequencing data of ancient human DNA (aDNA) to examine TEs in brain evolution.

## Transposable elements, a major evolutionary driving force

TEs are DNA sequences that can translocate or duplicate themselves within the genome and thus are abundant in human and NHP genomes. There are two major classes of TEs: DNA transposons, which mobilize in the genome through a non-RNA-mediated cut-and-paste mechanism, and retrotransposons, which mobilize through RNA-mediated copy-and-paste mechanisms. Among retrotransposons, there are long terminal repeat (LTR) retrotransposon families, including endogenous retrovirus (ERVs), and non-LTR retrotransposon families. We mainly focus on the latter, specifically LINE-1s (L1s), Alus, and SVAs (SINE-VNTR-Alus) that generate de novo copies at the rate of ~ 1/104 births, ~ 1/29 births, and ~ 1/192 births in human germlines, respectively [13]. We focus on these active human retrotransposons due to their potential relevance to recent human evolution after human-NHP divergence, such as during the Neolithic period. We recommend other reviews for detailed discussion on the evolutionary role of ERVs [14–16]. TE retrotransposition creates insertional polymorphisms, alters the copy number of existing genes, and sometimes creates insertion-associated genomic rearrangements [17], all contributing to a significant fraction of genomic structural variations (SVs) in the human population [18, 19].

TEs are large (*e.g.,* full-length L1s are 6 Kbp) and can affect gene function even when they are located in non-coding, intronic regions. For example, a primate-specific intronic Alu promotes RNA editing of a nearby exon coding for a GABA receptor and consequently lowers the excitability of the neuron [20]; another intronic Alu insertion in the *Fas* gene causes loss of the next exon in *Fas* mRNA, without affecting the splice junction, and results in autoimmune lymphoproliferative syndrome [21]. TEs contain transcriptional and splicing regulatory sequences and also promote the production of non-coding RNA (ncRNA), *e.g.*, microRNA (miRNA) and long non-coding RNA (lncRNA) [15, 22, 23]. To further replicate in the host genome, TEs, especially LTR elements, can retain promoters and *cis*-regulatory elements (*e.g.*, enhancers and insulators) abounded in binding sites for host transcription factors, though the binding sites in more ancient TEs may be no longer effective due to accumulation of neutral substitutions [15]. These elements within the TE sequences, together with TE-activated or TE-encoded miRNA and lncRNA, can regulate host gene expression in *cis* and in *trans*, during and after transcription [24], some of which have been known as vital for human brain development and neuronal protein synthesis (reviewed in [25]). Moreover, TEs with splicing donor sites (*e.g.*, the third open reading frame in primate-specific L1, ORF0 [26]) inserted in proximity to an exon with splicing acceptor sites can generate TE-exon fusion proteins, some of which are expressed in neurons (reviewed in [25]) and may contribute to human-specific brain features.

Multiple lines of evidence suggest the contribution of TEs to human brain evolution. Certain TE subfamilies (AluY, L1HS, SVA_E/F) expanded rapidly in the primate lineage during important periods of human brain evolution. This took place when the volume of the human brain tripled starting ~ 2–0.5 million years ago, and AMHs exhibited rapid behavioral changes about 50,000 years ago in Africa and the Near East [2, 25]. These expansions gave rise to ~ 1 million Alu, ~ 0.5 million L1, and 3000 SVA copies in the human genome [17]. This differentiated TEs in humans from those in closely-related NHPs [27, 28] and made TEs an abundant source of human-specific transcripts in the human brain [29] and potential human-specific transcription factor binding sites [30]. Importantly, genes expressed in neural tissues are generally long [31], making the brain more susceptible to splicing and expression-level changes induced by TE insertions [32]. Additionally, AMH-specific TE insertions annotated in the human reference genome are enriched in brain-related genes even after controlling for gene length [33].

Segmental duplications (SDs) [34], TE insertions, and other types of SVs have considerable impact on human and primate evolution [35–37]. TEs have undergone exaptation, where inserted TEs evolved, sometimes through post-insertional mutations, to confer phenotypes beneficial to the host survival, such as by acting as indispensable gene regulatory components in embryogenesis and innate immune responses (reviewed in [15, 34]). For example, the gene *GPR56* is involved in regional cerebral cortical patterning and has two noncoding exons homologous to a LINE and an Alu element [38]. TE retrotransposition can also create new genes by accidentally duplicating the flanking 5′ or 3′ sequences or can cause deletions of sequences close to the insertion sites [17]. TEs, especially from the two highly prevalent Alu and L1 families, can cause ectopic recombination through non-allelic homologous recombination, leading to chromosome rearrangements [17]. These TE-associated genomic events can quickly create new materials for evolutionary changes and contribute to the adaptation of organisms facing new environmental challenges [39]. Dysregulation of TEs may also contribute to the pathology of neurodevelopmental and neurodegenerative disorders (reviewed in [40–42]). Nonetheless, TEs are largely unexplored in existing human brain evolutionary studies due to the lack of reliable computational and statistical methods for large-scale TE profiling and evolutionary analysis in humans and NHPs.

## Comparative analysis of human and non-human primates

Comparative studies of human and other NHPs have reported several human-specific genetic, transcriptomic, and epigenetic changes related to brain function (Fig. 1A, B). The human-specific changes include previous SD events that generated the *SRGAP2* [43], *ARHGAP11B* [44], and *NOTCH2NL* [45–47] genes that are important for human brain evolution and other genetic elements, such as the transcription factor *FOXP2* [48]. Comparative studies have reported temporal changes in mRNA and protein expression [49] as well as lower levels of promoter methylation in the human prefrontal cortex compared to those in chimpanzees [50] (reviewed in [1, 8]). Most findings reflect changes that took place millions of years ago when humans diverged from NHPs and are thus limited in explaining human advances in behavior and culture thousands of years ago during the Neolithic period [51].

### Comparative genomic analysis

Comparative genomic studies identify variants under positive selection and are largely restricted to SNPs, SVs, and SDs [1] (Fig. 1A). Using SNPs, candidate positive selection regions are identified as genes with a large ratio of non-synonymous and synonymous changes of SNPs (Ka/Ks) in each coding region, *i.e.,* genes with Ka/Ks > 1 in humans but Ka/Ks ~ 1 in chimpanzees [52], and regions with low SNP diversity and excessive derived alleles [53, 54]. For SVs and SDs, positive-selection regions are detected by evaluating copy number differences between humans and chimpanzees [36, 55]. If a protein-coding TE insertion is under strong positive selection or in strong LD with the selected variant, the TE may carry SNPs with a large Ka/Ks ratio; regardless of protein-coding status, a TE insertion under strong positive selection may localize in a region of low sequence diversity [39]. However, TEs in protein-coding regions comprise a small portion of all TEs. These methods are also limited because a relatively small Ka/Ks ratio does not rule out the possibility of having SNPs in the codon of large effect size, and short read sequencing may be insufficient to resolve most evolutionarily recent repeats [1].

Several studies have investigated TE insertions under positive selection for insertions annotated in the reference genome (reference TEs) or those absent in the reference, *i.e.*, polymorphic in the population. Two studies identified species-specific TEs by comparing reference TEs in humans and seven NHP species [28, 56]. Consistent with a previous study of the TE insertion rate in humans and chimpanzees [27], these studies provide further evidence that the human genome has the largest number of insertions from recently active TE families, and that a considerable portion of species-specific TE insertions localize to genic regions, indicating high potential for TEs to influence gene function during human evolution.

Another comparative genomic effort investigated both reference and polymorphic insertions and deletions of Alu and L1 in 83 deeply sequenced NHP genomes released by the Great Ape Genome Project [57] and 10 additional modern human genomes [58]. The study showed that phylogenetic trees and the Principal Component Analysis (PCA) of different individuals based on their polymorphic TE insertions capture their evolutionary relationship, largely consistent with the results from SNPs. However, limited benchmarking of polymorphic TE detection tools, especially for NHPs, makes it difficult to integrate TEs for a comprehensive cross-species evolutionary inquiry [59].

Other types of sequence-level changes, notably those in Human Accelerated Regions (HARs) [60] and human-specific sequence losses have been examined. HARs are defined as genomic loci conserved among other species but with elevated divergence in humans. The rationale for including HARs in evolutionary studies is that their conservation in multiple species suggests their functional importance, and that they contain human-specific changes that can contribute to advanced human social and cognitive behavior [60]. Khrameeva and colleagues [61] identified evolutionarily important genetic variants based on their overlap with or proximity to the HARs curated by Vermunt et al. [62]. A study of human-specific deletions highlighted the contribution of regulatory DNA, especially tissue-specific enhancers, to human brain evolution [63].

Long-read sequencing technology, such as PacBio and Oxford Nanopore, have refined primate genome assemblies, usually in repeated regions and haplotypes [60], and enabled comparative genomic studies of complex variants, including SVs, SDs, short tandem repeats (STRs), and TEs [35]. Long-read sequencing can detect complex variants more effectively because the reads can fully cover the repeat sequence and resolve mapping ambiguity to the reference genome, a common issue in short-read sequencing [64]. With rapidly evolving genome assembly methods, long-read sequencing has even enabled the reference-free discovery of complicated genetic variants. For example, a 2017 study generated long-read sequencing data of haploid bacterial artificial chromosome (BAC) clones for hundreds of SDs including genes in humans and NHPs and annotated more SDs in the human reference genome based on alternative sequence assembly [36]. With improved assembly, the authors performed joint analysis of published short-read sequencing data of diverse modern humans [18, 65], archaic humans [66, 67], and NHPs [57] using multiple sequence alignment and paralog-specific read mapping. They identified three SD-embedded genes with low copy-number polymorphisms in modern humans that have specifically expanded in modern humans compared to archaic humans. A study using long-read sequencing and linked reads resolved STRs in several NHPs and highlighted the potential impact of STRs on brain evolution [68]. Intriguingly, the study showed that active SVA retrotransposition is largely responsible for human-specific STR expansions.

Advances in comparative genomics include incorporation of more NHPs and improved genome annotations of all primates. Following the Great Ape Genome Project [57], scientists have extended their search for human-specific genetic changes within human-chimpanzee-mouse cross-species genomic comparisons to human and multiple other closely related NHPs [35, 61], with more distantly related monkeys (*Rhesus macaque* and *Callithrix jacchus*) as primate out-groups [8]. WGS datasets of multiple representative individuals from many NHP species help refine the annotations (orthologous genes and non-coding regions) of NHP genomes, which in turn facilitates cross-species comparisons of many more genetic variants on a larger fraction of genomes [35] and applications of genome-wide comparative methods computationally tailored for the data [69].

The ongoing active retrotransposition of TEs can generate more recent genomic changes contributing to human brain evolution. As limited differences have been observed within protein-coding genes between human and chimpanzee [35], cross-species transcriptomic comparisons may unveil human-specific gene regulatory differences contributing to higher brain functions. By leveraging comparative genomic strategies applied to other genetic variants and improved genome annotations of humans and NHPs, scientists may be able to link many more individual TE insertions to changes in chromatin structure and brain-related gene expression.

### Comparative transcriptomic analysis

Comparative studies transcriptomic analyses of human and NHP brain tissues and cells have unveiled multiple unique aspects of the human brain (Fig. 1B). The human brain has been shown to have higher gene expression levels than that of closely related NHPs and exhibit larger transcriptomic complexity than other tissues, potentially explaining higher neuronal activity and synaptic plasticity conducive to human brain evolution (reviewed in [70]). Moreover, non-coding transcripts in the brain tissue, including additional introns, intergenic repeats, long and short ncRNA, some of which are encoded by Alus and other TEs, can orchestrate complex spatiotemporal gene regulatory programs unique in the human lineage (reviewed in [24, 70]). Cross-species differences in alternative splicing are highly prevalent in the human brain [71, 72] and have been strongly associated with psychiatric diseases [29, 73, 74].

TEs may contribute to the uniqueness of the human brain by providing alternative splice sites [22], causing different proportions of splicing variants between humans and NHPs. Alu-containing exons are present in a substantial fraction of major mRNA splice isoforms in the human brain [75], but it is unclear whether this leads to significant evolutionary consequences. We review findings of differentially expressed TEs, followed by rationales and challenges of existing computational methods for comparative transcriptomic studies. Since most biological results were derived from studies of non-TE genetic elements, we focus on methods that are adoptable to analyzing protein-coding TEs as well as assessing the expression level changes of host genes regulated by non-coding TEs.

Most comparative transcriptomic analyses have focused on reference TEs and have identified differential TE expression across species, tissue types, and developmental time points. Primate-specific ERV and L1 are highly expressed early in development and have undergone exaptation to regulate the expression of lncRNA and host genes, influencing blastocyst development, stem cell pluripotency, and antiviral resistance (reviewed in [14, 15, 34]). Interestingly, the same transcriptional dynamics have been observed for rodent-specific ERVs in mice (reviewed in [15, 34]). In particular, short interspersed nuclear elements (including Alu and other closely related TE families), can function as crucial enhancers during mammalian brain development [76]. Moreover, TE expression is highly regulated and variable in different human tissues and cell types, especially for L1 and ERV [77–79]. A cross-species study reported increased expression of *APOBEC3B* and *PIWIL2*, two genes involved in the restriction of L1 retrotransposition, in human compared to NHP induced pluripotent stem cells (iPSCs), suggesting a role of L1 mobility in shaping primate genomes and continuing adaptation [80]. Further studies are necessary to determine whether groups of tightly regulated TE expression execute coordinated functions during human-specific brain development, hypothetically through regulations of gene expression and chromatin accessibility and independent of the mobility of TEs [34].

In multiple organisms, TEs can induce rapid spatiotemporal changes in response to environmental cues, both during and beyond embryonic development, by changing TE expression and/or modulating the host genes under TE promoters (reviewed in [14, 24, 34, 81–83]). Following this logic, numerous additional (exapted) copies of TEs in the human genome, present in over one-third of the human protein-coding transcripts and three quarters of human ncRNA [84, 85], confer an extra layer of plasticity in gene expression and can contribute to humans’ ability to adapt to environment [83].

TE-binding Kruppel-associated box (KRAB) zinc finger proteins (KZFPs), transcriptional silencers of TE families including ERV and L1 [86], have contributed to human-specific regulatory network in human neurons [87, 88] and led to differential expression between human and chimpanzee [89]. Rather than completely silencing transcription of TEs in embryogenesis, KZFPs such as ZNF417 and ZNF587 (absent in mouse) control regulatory sequences consisting of exapted TEs, and consequently affect expression of hundreds of human genes in developing and adult human brain [90]. As an unexpected outcome from the evolutionary arms race to repress expression of evolving L1 sequences, a KZFP transcription factor ZNF558 (highly expressed in human but not in chimpanzee forebrain neural progenitor cells) has been exapted to repress mitophagy and potentially contribute to human-specific cortical expansion [91]. More examples of TE and KZFP’s contribution to species-specific mammalian development are recently reviewed by Senft and Macfarlan [14].

Lanciano and Cristofari have reviewed experimental and computational tools to quantify genome-wide TE expression levels as well as the associated challenges [92], which will be instrumental for expanding comparative transcriptomic analyses to TEs. To fully appreciate TEs’ impact on human transcriptomes, it is critical to analyze the expression of polymorphic TE insertions generated by relatively young TE families [92], as these sites are expected to preserve most of the binding sites for regulatory elements [93] and some are under recent natural selection [94, 95]. However, determining locus-specific expression level of polymorphic TEs remain challenging, because of the low mappability of young TE sequences and the confounding expression of nearby host genes, as opposed to autonomous TE expression [92]. Tools such as *NearTrans* [96] and *TEffectR* [97] have been developed to identify differentially expressed TEs and associate them with differentially expressed genes (DEGs) or nearby genes, respectively in the context of cancer, and the methods can be potentially repurposed to identify candidate regulatory TEs in the context of evolution.

To measure the impact of TEs on transcriptomes, it is beneficial to borrow strategies from comparative transcriptomic studies of brain-related genes. Cross-species comparative transcriptomic studies compare brain transcriptomes from different brain regions (spatial comparison) at different time points of development (temporal comparison) using heterogenous metrics of species specificity. The spatial comparison assesses the correlation of gene expression levels for the same brain regions of different species using generic differential expression analysis tools [98], linear models with species as a covariate [61, 99], and unsupervised hierarchical clustering [61, 99, 100]. Studies have also examined global gene expression patterns by identifying modules of genes with similar variation across brain regions and/or species, for example, using Weighted Gene Co-expression Correlation Network Analysis (WGCNA) and PCA-based gene ontology analysis [74, 98, 100]. For temporal comparison, the effect of developmental age across species has been taken into account using Gaussian Process-based models, *TranscriptomeAge* and *TempShift* [74].

To decipher the relevance of species-specific expression-level changes to brain evolution, many studies have explored DEGs between humans and NHPs using existing gene ontology annotations and external datasets in brain development and diseases. Specifically, using DEGs, studies have performed functional enrichment tests, relating regulatory elements to known evolutionarily important variants [61, 98], and conducting transcriptomic signature analysis [74]. Based on knowledge of temporal transcriptomic change during brain development [61], one study predicted downstream phenotypes using genes associated with human-specific neurological and psychiatric disorders [74]. When the DEGs encode transcription and epigenetic factors, published epigenomic data were integrated to predict affected downstream pathways [74, 98, 100]. However, most studies have only focused on correlating expression-level changes with expansion of the neocortex and have provided indirect information on how affected cell types, genes, and/or proteins are related to changes in cognitive function [101].

Comparative transcriptomic studies use a wide range of RNA (mRNA, miRNA, lncRNA) annotation strategies. They include using default gene annotations within the reference genome assembly [61] with genomic coordinate conversion tools [98], using other published sequencing results for guidance [99], and employing a computational framework specifically designed for ortholog annotation across primates (*e.g.*, the XSAnno pipeline [69, 74, 100]). Since ncRNA may not be clearly associated with annotated gene(s), but can play a regulatory role in *cis* and in *trans* (reviewed in [102, 103]), one study reannotated miRNA of NHP samples guided by human miRNA precursors [100]. A unified gene and RNA annotation framework would be important for a comprehensive investigation of cross-species RNA expression differences. Furthermore, given the different quality of orthologous region annotations in primates and the high cost of transcriptome profiling of primate brains, it may be advantageous to develop cross-species data integration tools that enable large-scale analysis to draw meaningful conclusions, as in the case of gene annotation [69]. As the impact of a TE insertion largely depends on its insertion locus [77], *e.g.*, proximity to brain-related genes, and in some cases its ability to drive ncRNA expression [15, 24, 25], we raise caution with RNA annotation when conducting TE-related comparative transcriptomic analyses.

A comparative transcriptomic approach is significantly limited by the scarcity of brain sample sources. Using post-mortem brain tissue suffers from RNA degradation and can yield biased transcriptome quantification depending on many pre- and post-mortem factors [70]. Given that the primate brain is largely inaccessible and has highly dynamic transcriptomes that vary throughout the primate lifespan, primate brain region-specific organoids derived from iPSCs of individuals at all life stages are promising tools to recapitulate the spatiotemporal changes during brain development, including those in the formative prenatal periods [70, 104, 105]. However, in addition to modulating host gene expression and diversifying transcript isoforms, TEs can also alter mRNA localization and stability, translation efficiency, and the epigenetic landscapes of nearby regions (reviewed in [15, 24, 34]), which would require further techniques and analyses.

## Population genetic approaches to identify variants under selection

To identify evolutionarily important genetic variants, including TE insertions, population genetic approaches utilize large-scale variant sets across diverse humans representative of the entire species (Fig. 1C) and powerful statistical methods to narrow down variants under positive selection (Fig. 1D) or involved in polygenic adaptation (Fig. 1E). We review genome-wide positive selection scan methods adaptable for TEs as well as consortium efforts that have released WGS datasets essential to perform genome-wide positive selection scans for TE insertions. Since human brain evolution is manifested in many polygenic traits [106–108], we also review recent advances in polygenic adaptation scans and discuss the possibility of incorporating TEs into these computational frameworks.

### Positive selection scans

Most publications from large consortia of modern humans have conducted simple positive selection scans to identify variants located in genomic regions under positive selection. Both the 1000 Genomes Project (1KGP) and Sudmant et al. 2015 focused on SVs and CNVs with significant variation in AFs across continental populations. Among the population-stratifying SVs, Sudmant et al. identified two CNVs that have been associated with cognitive functions, autism severity, anxiety, and neurotransmission functions [18, 65]. Similarly, a study from the Human Genome Diversity Project (HGDP) reported SVs with significantly higher population differentiation based on pairwise population AF comparisons and high population branch statistics scores [109]. The gnomAD-SV team developed the Adjusted Proportion of Singletons (APS) metric to determine the strength of natural selection based on the proportion of ‘singletons’, *i.e.,* SNPs present in only one allele in the population, and detected negative selection in almost all gene-altering SVs [110]. However, when applying simple selection scans to diverse modern and ancient populations, false positives can result from population stratification—the presence of systematic differences in allele frequency across different populations—and other demographic processes [111–114].

The Simons Genome Diversity Project (SGDP) conducted a more sophisticated positive selection analysis by taking demographic confounders into account, using pairwise sequentially Markovian coalescent [115], the 3-population composite likelihood ratio [116], and other methods. The study provided evidence against the hypothesis that human brain evolution was caused by a few genes under strong positive selection [10]. Davis et al. have also failed to find strong selective sweeps within SNPs associated with brain and behavioral phenotypes using both long-range haplotype-based tests and population differentiation tests [117]. These failures call for development of more powerful genomic scans that are applicable to other portions of the genome, such as TE sequences, and for development of other genetic models tailored to detect variants that existed before the onset of positive selection. WGS data from multiple consortia have become abundantly available for further TE profiling, for example one study has profiled TEs in diverse modern humans, archaic humans, and chimpanzees [118].

The TE insertion profiles of modern humans can help interpret TE insertions in aDNA to study brain evolution; however, very few positive selection scans have been adapted for TEs in humans. Most TE insertions are under negative selection and have very low AF because they likely reduce host fitness by disrupting gene sequences and regulatory elements [119]. A rise in AF of a TE insertion does not guarantee that the insertion is under positive selection since a high AF could result from genetic drift for small populations and gene flow from migration and interbreeding of genetically divergent populations. Therefore, a recent study of positively selected TE insertions in 1KGP samples yielded a null genetic model that used the effective population size and timing of population divergence to control for demographic history. It revealed six TE insertions with unusually differentiated AFs between populations as evidence of positive selection, but none of them were linked to human brain evolution [119]. This null genetic model has thus been criticized as oversimplified [39] and lacking statistical power [119]. This failure also suggests the need for a polygenic adaptation model where variants are combined to explain the phenotype when AF changes in individual variants are otherwise too subtle to be detected.

Genome-wide positive selection scans over different time scales within modern humans (reviewed in [120]) can be adapted to jointly analyze TE insertions and their nearby SNPs in the same individuals [39] (Fig. 1D). When a TE insertion is under positive selection, its AF will increase and elevate the AFs of SNPs linked in the same LD block, forming a region of low sequence diversity as an indication of positive selection. The selection can be detected by Tajima’s D test [53] (an allele frequency spectrum test) and other long-range haplotype-based methods [121]. Using SNPs also better controls for demographic history and recombination rates that affect the TE insertion rate [39, 122]. The AFs of a recent TE insertion and nearby SNPs may increase before recombination breaks up the local haplotype structure, leading to an abnormally extended haplotype, which can be detected by the Cross Population Extended Haplotype Homozygosity (XP-EHH) test [121]. XP-EHH is applicable to highly differentiated populations and shows promising identification of TEs under positive selection in flies [94] and SNPs in humans [121]; however, it has not been implemented for TEs in humans. While implementing these joint SNP-TE approaches can facilitate a more comprehensive null genetic model for TEs, the possibility of TE insertions under partial or soft sweeps also needs to be considered [39].

### Polygenic adaptation scans

Many human traits are under the influence of many small effect genomic loci, *i.e.,* polygenic [107]. Under polygenic adaptation, each contributing individual locus will have a subtle shift in AF that cannot be detected by previously reviewed positive selection scans. Polygenic adaptation scans developed for polymorphic SNPs in modern humans can inform the development of population genetic models of TEs under polygenic adaptation (Fig. 1E). There are two categories of polygenic adaptation scans: those detecting trait-associated alleles with correlated AF changes or those detecting selection-induced distortion within the genealogy tree of the population of interest.

Since polygenic adaptation involves subtle but correlated changes in AFs of trait-associated alleles with small effect size, several methods aim to identify positive covariance between the AF changes of these alleles, achieving more statistical power than single-locus-based positive selection scans [123–129]. For example, Berg and Coop estimated genetic values for each complex trait in each population using the linear weighted sum of the AFs of alleles positively associated with traits, where the weight was proportional to the effect size of an allele in the genome-wide association study (GWAS) of the matching population [123]. The genetic values were tested to see if they showed higher covariance among the tested populations than would be expected from genetic drift or shared ancestry alone. This method can be applied to any complex trait, as long as GWAS effect size estimates are available for the matching population. This approach can detect polygenic adaptation occurring up to ~ 30,000 years ago according to data simulations [123]. Using this method, Davis and colleagues observed polygenic adaptation of the following traits: schizophrenia, extraversion, subjective well-being, structure volumes of brain regions including hippocampus and putamen, and other immune diseases [117].

The second group of polygenic adaptation scans include the Singleton Density Score (SDS) test and the trait SDS (tSDS). The methods assume that a recent selection event occurring up to the 2000 years ago would rapidly increase the frequency of an allele before new mutations randomly occurring in one person started to accumulate in the same haplotype within the population [130]. Therefore, recent selection would lead to a larger interval of surrounding singleton SNPs, *i.e.,* low SNP density, within the entire sample population and consequently shorter terminal branch lengths within the genealogy tree of individuals constructed using SNPs (Fig. 1E). Using this approach, Davis and colleagues found evidence not only of positive selection in increased total intracranial volume but decreased subcortical brain volume, schizophrenia-protective alleles, and height-increasing alleles, but also of negative selection in Type 2 diabetes-protective alleles [117]. Similarly, the Polygenic Adaptation Likelihood Method uses sampling from a genealogy tree and corresponding GWAS summary statistics to search for targets of very recent polygenic adaptations [131]. While Davis and colleagues have provided valuable insights into the impact of natural selection on human brain evolution, these polygenic adaptation scans have mostly been used on homogeneous modern European populations, which may not represent the entire human species. Furthermore, applying the methods to diverse aDNA will require significant modification of the methods and accurate TE insertion genotyping in a large number of ancient individuals.

In order to establish causal relationships between genotype changes and enhanced brain functions, genetic variant candidates uncovered from population genetic approaches need to be experimentally validated in mechanistic studies to gain further biological insights. It is possible that variants with indications of strong natural selection can only have minor effects on phenotypes implicated in brain evolution [15].

## Time series ancient human genome analysis to identify variants under selection

**Fig. 2:**
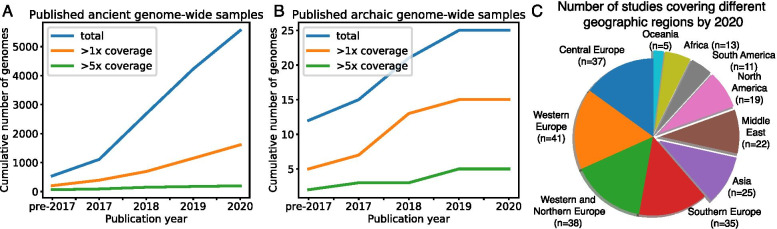
Statistics of available ancient/archaic human genomic data. The cumulative numbers of ancient (**A**) and archaic (**B**) human samples profiled with WGS and genome-wide SNP arrays are shown according to publication years. Blue, orange, and green lines indicate the number of total WGS datasets, and those with >1x and > 5x coverages, respectively. (**C**) The number of ancient human studies covering different geographic regions by 2020. Non-European regions are popped out in the pie chart

Recent technological advances in ancient/archaic DNA extraction and sequencing have enabled large-scale production of genome-wide ancient and archaic human datasets covering wider spatiotemporal ranges spanning key periods of human brain evolution. As of the end of 2020, genome-wide datasets (WGS and SNP arrays) have been available for 5560 ancient human individuals as well as for 25 archaic human individuals (Fig. 1F; Fig. 2; Additional file 1: Table S1–2). Notably, there are WGS datasets that have allowed TE genotyping of five archaic human individuals and 198 ancient human individuals sequenced at >5x coverage (Fig. 2A–B; Additional file 1:Table S1–2). While ancient humans living in Western Eurasia over the past thousands of years have been heavily sampled, 67 studies have thus far included samples outside the Western Eurasia continent [51] (Fig. 2B), better representing globally dispersed AMHs. Many of these samples originated from humans before or during the Neolithic period, when the human society was transitioning from nomadic hunter-gatherers to agricultural settlements with gradual development of unique human culture, agriculture, and animal domestication [51], and thus may allow unprecedented sensitivity to capture the underlying genetic causes, if any [106].

Large aDNA datasets provide unique opportunities for human evolution studies and may continue to alter pre-existing conceptions and resolve controversies [51, 106]. aDNA may contain information explaining non-skeletal human phenotypes and behaviors not preserved in archaeological records [132], and allowing selection events to be inferred from more than thousands of years ago [130, 133]. For example, Mathieson and colleagues detected selection loci associated with diet, skin pigmentation, and immunity, and resolved two episodes of selection on height using a positive selection scan through aDNA genotype data of 230 ancient Eurasians from 6500 to 300 BC [124]. The genetic homogeneity of aDNA from 174 Caribbean individuals who lived ~ 2500 years ago discredited the previously hypothesized influx of genetically different populations underlying changes in pottery styles and supported another argument that the style changes stemmed from communications within Caribbean populations [134]. Moreover, aDNA has ended the debate over the origin of modern humans by offering definitive evidence that ancient African AMHs contributed to the majority of modern human genomes [7, 106, 135].

Many genetic discoveries have been made by comparing a small number of archaic human genomes with modern human and NHP genomes, but the studies have rarely focused on brain evolution or TEs. On one hand, segments of archaic genomes introgressed into modern human populations via interbreeding are under positive selection [55, 114] and have been instrumental in tracing ancient human migrations and admixtures [65, 136, 137]. On the other hand, evolutionary conclusions drawn from modern human and NHP genomes have been validated in only a limited number of archaic genomes. For example, a few archaic human genomes were analyzed with other modern human genomes to identify SDs expanded in AMHs [36], CNVs duplicated in the human lineage [138], and differentially methylated regions in AMHs [139]. To date, the only TE study in human brain evolution analyzed species-specific reference TE insertions in two archaic human genomes, modern humans, and chimpanzees [33]. The study identified a trend of enrichment of human-specific TEs in genes expressed in brain tissues, warranting follow-up studies with more comprehensive TE profiling, AF tracking, and validation of the impact on brain-related phenotypes. The wide spatiotemporal coverage of increasingly available aDNA sequences will allow us to trace changes in TE insertion AFs in ancient humans and identify evolutionarily important TE insertion candidates. In order to gain insights into how these TE insertions contribute to human brain evolution, we can correlate them with linked SNPs implicated in GWAS of brain-related traits.

Efforts to decode past human migration patterns from aDNA provide valuable resources for brain evolution studies. Methods to infer selection based on time-series AF data developed for aDNA [140, 141] can be applied to or adapted for TE-related natural selection scans. These studies also provide demographic information of ancient humans, such as population migration, mixture, and structure [132, 142], that is crucial for proper interpretation of AF changes and creation of population genetic models incorporating confounding factors such as genetic drift and gene flow.

## Technical challenges in TE insertion profiling with ancient DNA

**Fig. 3:**
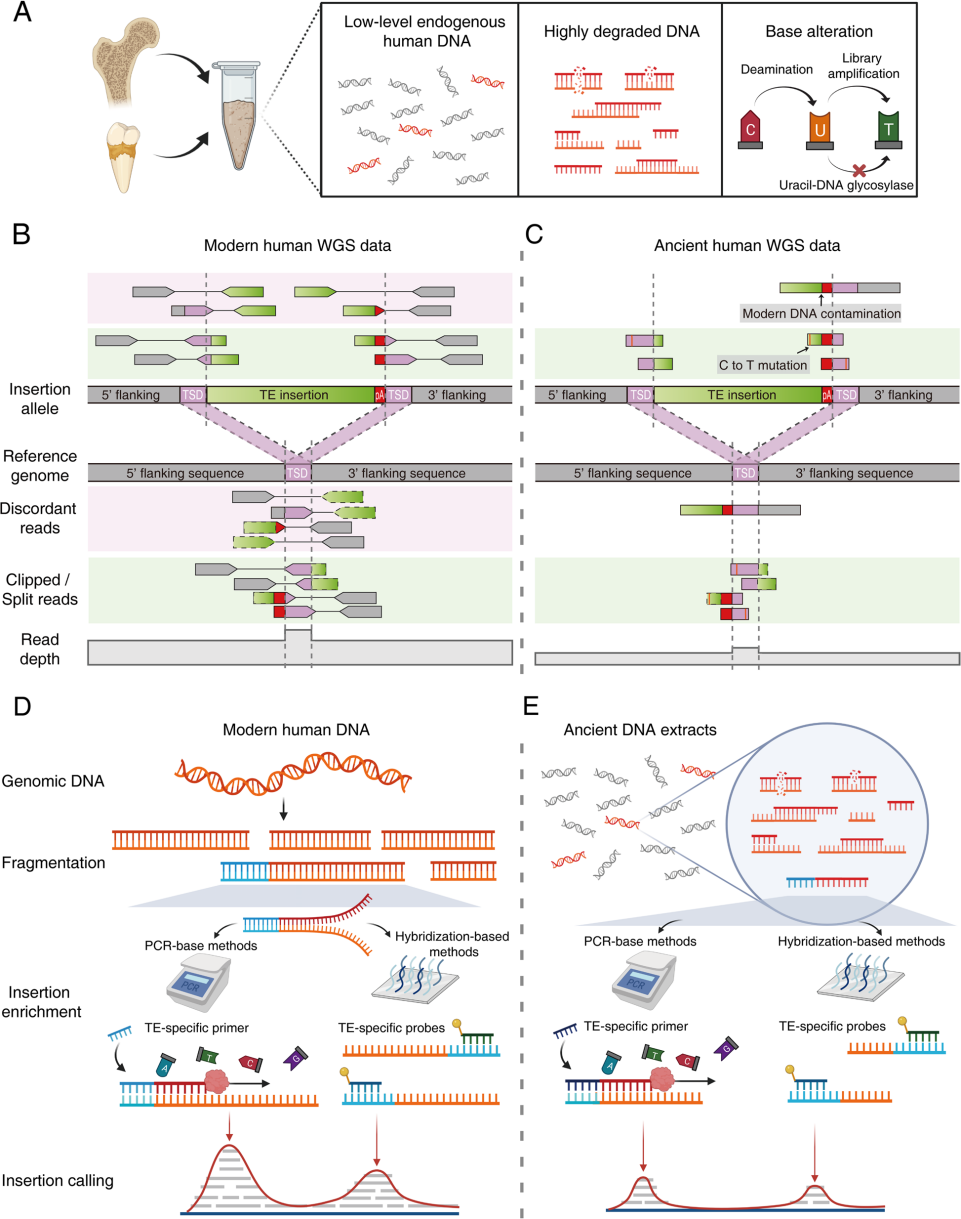
Technical challenges of TE insertion profiling for ancient samples. (**A**) Common features of DNA extracted from ancient humans: 1) Ancient human samples have low-level endogenous aDNA due to DNA contamination; 2) Small amounts of highly degraded aDNA are preserved in ancient bones, teeth, and sediments; 3) Cytosine deamination is a hallmark of base damage in aDNA, resulting in C to T substitution in sequencing data. Uracil-DNA glycosylase (UDG) treatment can be applied to aDNA in pre-library preparation to reduce the base error. (**B**) WGS-based TE insertion profiling using specialized bioinformatic tools. In modern human WGS data, paired-end reads derived from a polymorphic, non-reference TE insertion are aligned to the reference genome. Tools mainly detect two types of reads near the insertion breakpoints: i) discordant reads (light pink box) that are uniquely aligned to the flanking regions and have their mate-pair reads aligned to many reference TE copies remotely located from the breakpoints, and ii) clipped reads or split reads (light green box) that span the insertion breakpoints and thus have soft-clipped or split mapping to the reference. A read-depth increase due to target site duplication (TSD) is shown. Grey dashed lines indicate the boundaries of TSDs. (**C**) Characteristics of ancient human WGS data pose challenges in TE insertion detection: limited sequencing depth, short read length, single-end mapping, prevalent C to T substitutions enriched in DNA fragment ends, and occasional contamination of modern human DNA. (**D**) TE insertion profiling using targeted TE-capture sequencing. Common TE-capture sequencing steps include DNA fragmentation, TE-junction enrichment, next-generation sequencing, read alignment, and insertion calling. DNA fragments originating from TE-junctions are captured and enriched by PCR using TE-specific primers or hybridization using TE-specific probes or microarray. (**E**) TE enrichment with aDNA extracts is challenging due to a limited amount of endogenous human DNA and highly degraded DNA fragments

Over the last three decades, researchers have made remarkable technological advances in extracting DNA from highly degraded samples in ancient remains [143–145] and creating ancient DNA libraries for high-throughput sequencing [146–148], replacing earlier methods of molecular cloning followed by Sanger sequencing [149]. There are two different approaches for genome-wide TE insertion profiling with ancient DNA: using bioinformatic pipelines designed for WGS data [33, 118] and performing targeted TE capture sequencing. However, unique characteristics of aDNA samples pose great technical challenges for both approaches (Fig. 3A). First, aDNA samples have a low percentage of endogenous ancient human DNA due to substantial microbial and environmental DNA contamination, which leads to a large waste of sequencing throughput. Second, aDNA samples are highly degraded, resulting in short DNA fragments in aDNA sequencing libraries. Third, aDNA has frequent sequence alterations due to cytosine deamination, which could be partially eliminated using Uracil-DNA glycosylase (UDG) treatment in library preparation [150]. Lastly, scientists should remain vigilant to accidental contamination of ancient human samples by DNA from modern human researchers or modern humans inhabiting the same geographic region as ancient humans [51, 106]. Such contamination can be detected by an unusually high fraction of long sequencing reads [151] and/or be estimated by the fraction of sequencing reads with DNA damage signal (C to T substitutions) toward the ends of reads [152, 153] (see [132, 142] for approaches for contamination correction, along with [154, 155]).

Most WGS-based computational TE insertion detection tools designed for modern human DNA rely on two types of anomalous reads in paired-end sequencing data: discordant read-pairs and clipped reads that indicate non-reference TE insertions [156] (Fig. 3B). At the breakpoints of non-reference insertions, discordant reads are aligned to the flanking regions, with their mate-pair reads remotely mapping to TEs. Meanwhile, clipped reads span TE-junctions with their TE sections soft-clipped. Utilizing sequence information from the two types of read clusters supporting a TE insertion, the genomic locations and features of TE insertions could be characterized, including insertion size, orientation, target-primed reverse transcription (TPRT) hallmarks (target site duplication (TSD), polyA tail, L1 endonuclease cleavage motif), and genotype.

Application of the WGS-based methods to ancient human WGS needs to overcome multiple technical challenges (Fig. 3C). First, most ancient WGS data have limited sequencing depth due to sample contamination and sequencing cost [157] (Additional file 1: Table S2). The limited sequencing depth decreases the accuracy of TE detection and genotyping. Second, aDNA templates are highly fragmented, and shotgun WGS libraries are sometimes sequenced by the single-end mode with short read length [33, 158]. For libraries sequenced by the paired-end mode, read pairs largely overlap with their mate reads. Thus, aDNA WGS analysis pipelines typically merge the paired-end reads into single-end contigs for a higher mapping rate and more accurate alignment [159]. This merging process necessitates an extra read unmerging step to utilize existing paired-end TE detection tools. Finally, deaminated cytosine residues that are enriched at the ends of aDNA molecules may affect TE subfamily classification based on the diagnostic SNPs within the TE sequences [146].

For cost-effective genome-wide TE profiling, many TE-targeted sequencing methods have been developed [160–167]. These methods have a similar workflow: DNA fragmentation by sonication or enzymatic digestion, TE-junction enrichment by TE-specific PCR primers or TE-specific probes followed by next-generation sequencing, and TE insertion calling based on the read alignment of the TE flanking sequences (Fig. 3D). Several targeted sequencing methods have been developed to detect low-level mosaic TE insertions present only in a small number of cells from bulk DNA [164, 166, 168, 169]. These sensitive methods may have the potential to capture TE insertion signals from aDNA extracts effectively (Fig. 3A). Since TE families relevant to human brain evolution are likely to be primate-specific, TE-targeted sequencing can eliminate microbial DNA without sacrificing the yield of endogenous DNA through enriching TE-junction sequences. Considering the short length of aDNA templates, TE-specific primers and probes need to be optimized to hybridize close to the end of TE sequences so that the junction flanking sequences can be effectively amplified and sequenced (Fig. 3E).

## Functional validation of evolutionarily important TEs

**Fig. 4:**
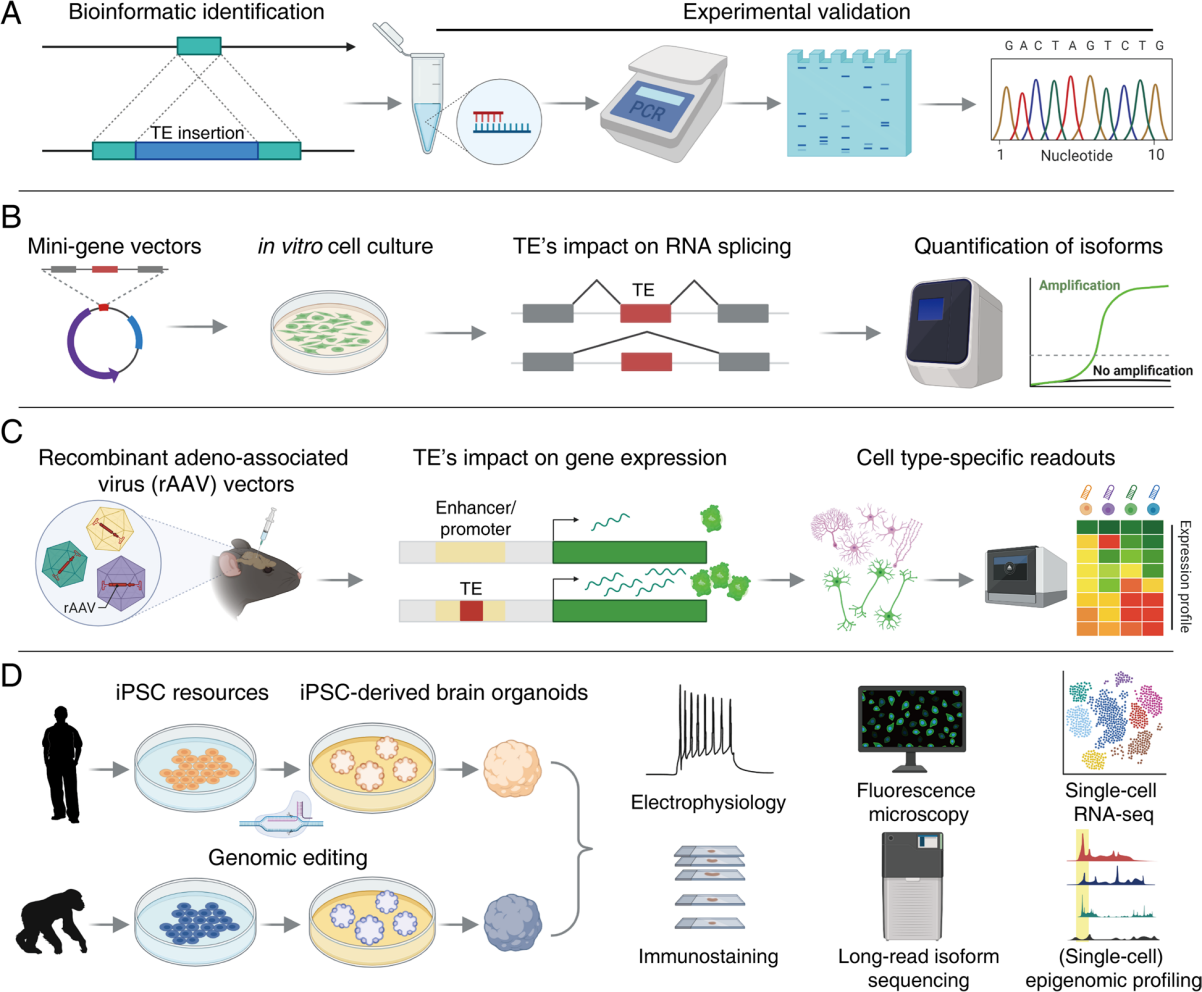
Characterization and functional validation of TE insertions. (**A**) Bioinformatic identification and experimental validation of TE insertions. Putative TE insertions are validated by PCR and Sanger sequencing. Alternatively, insertions can be computationally confirmed and reconstructed using modern human short- and long-reads sequencing data. (**C**) Investigating functional impact of TE insertions. TE insertions can be cloned into the mini-gene and recombinant adeno-associated virus (rAAV) vectors to assess their impact on RNA splicing in vitro and gene expression in vivo, respectively. (**D**) Understanding the role of TE insertion in brain development. Recent advances in cellular and organoid models, genomic editing technologies, and single-cell multi-omics sequencing allow for the investigation of TE’s functional impacts by characterizing phenotypic changes and dissecting molecular changes in gene expression, splicing, and epigenetic states in a well-controlled culture environment

TE insertions are a significant source of genetic and transcriptional variations in species [170]. TE insertions exert functional impact on target genes through various mechanisms, such as altering gene expression and RNA splicing, creating or disrupting regulatory elements (e.g., enhancers and promoters), and promoting genomic rearrangement via homologous recombination and exon shuffling [171]. We review experimental strategies and considerations to validate candidate TEs identified by aforementioned analytical approaches. Because of the multicopy nature of TEs—the human genome bears additional copies of similar repetitive sequences, uniqueness or sequence specificity of candidate TE insertion region needs to be warranted in both experimental design and data analysis. Generally, TE insertions are validated by PCR of the insertion allele using primers that amplify the entire TE with proximal flanking sequences, or 5′ and/or 3′ junctions of the insertion (Fig. 4A). Sanger sequencing of the PCR product allows further characterization of the insertion, which facilitates the understanding of the impact of the insertion. The validated TE insertion can be cloned and studied with the minigene assay to evaluate the insertional effects on RNA splicing and expression in vitro [22, 172] (Fig. 4B). For in vivo assays, it is desirable to validate TE insertions simultaneously in a scalable manner [173]. To probe insertions’ impact on different *cis*-regulatory elements, a barcoded-pool of recombinant adeno-associated virus (rAAV) library can be constructed and injected to the target tissue. Cell type-specific expression of different barcoded vectors can be determined by single-cell RNA sequencing (Fig. 4C). Because of packaging capacity of AAV, vectors with candidate TEs are limited to 5 kb in length, which is not suitable for large TE insertions (*e.g.*, full-length L1). Finally, recent advances in editing and manipulating the genome and the epigenome allow for the investigation of functional contribution of an entire TE (sub) family [174, 175].

Understanding the impact of candidate TE insertions on human brain development requires models that permit genetic manipulations to recapitulate the role of TEs during different developmental stages (Fig. 4D). For example, CRISPR/Cas9 excision of a pathogenic TE insertion in patient iPSCs rescued molecular phenotypes in neural stem cells and differentiated neurons [176]. iPSCs and neuronal progenitor cells (NPCs) from chimpanzee and other primates have been generated to probe cellular and molecular differences between human and NHPs [80, 91]. Leveraging recent innovations in iPSC-derived brain organoids for 3D modeling, researchers can investigate TE insertion-induced phenotypical changes during early cortical development [177]. Due to nearly identical TE copies, especially for young TE families, it has been challenging to distinguish the expression of active TE loci from pervasively transcribed inactive TE copies. Long-read sequencing technologies enable the detection of TE-derived transcripts in a locus-specific manner [92, 178]. As multiple studies have recently demonstrated, single-cell RNA-seq and accessible chromatin profiling of genetically manipulated brain organoids will allow us to analyze epigenetic changes, activity states, and functional effects of TE insertions [179, 180]. In summary, with recent technical advances in brain organoid models, long-read sequencing, and single-cell multi-omics approaches, we have an unprecedented opportunity to assess the effects of evolutionarily important TE insertions on human brain development and phenotypic divergence.

## Conclusions and future perspectives

We have reviewed computational and experimental approaches to identify and validate evolutionarily important genetic variants, especially for TEs in human brain evolution. We have also highlighted strategies that leverage ancient genomic data and discussed unique challenges in ancient transposon genomics. Comparative and population genetic studies have shed light on many unique genetic features of the human brain, but more experimental and computational investigations are warranted. Rapidly increasing genomic datasets of modern humans, NHPs, and ancient humans offer a unique opportunity to systematically evaluate the evolutionary role of TEs in shaping human brain function. While ancient human datasets provide snapshots of human genomes in the past and enable powerful time-series analyses for brain evolution inquiries, aDNA is susceptible to degradation, contamination, and DNA damage over time, which inevitably poses technical challenges to overcome for accurate TE insertion detection and genotyping. Thus, research in bioinformatics, evolutionary biology, and neurobiology is necessary to study the role of TEs in human brain evolution.

A large amount of WGS and high-density SNP genotype data from modern humans may allow the creation of SNP-TE haplotype reference panels. The panels may allow the imputation of unobserved or un-genotyped TE insertions that are present at a certain AF within the modern human populations, even in ancient humans with only SNP data. Thus, we can create a high-quality TE insertion map from ancient human samples by capitalizing on the combination of computational, experimental, and statistical inference approaches. Future efforts should focus on sequencing more diverse ancient humans and developing better computational tools for TE insertion detection and statistical models for TE population genomics.

**Fig. 5:**
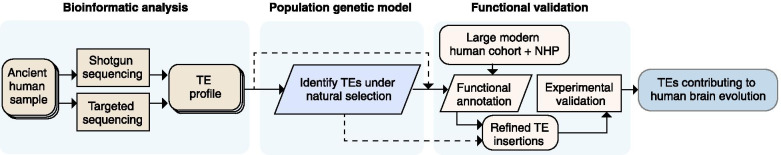
Leveraging ancient human genomes to understand the role of TEs in human brain evolution**.** First, profile polymorphic TE insertions in a large number of diverse ancient human genome sequencing data. Second, derive population genetic models to detect TEs under natural selection and/or perform functional annotation of TE insertions to create a refined list of polymorphic and reference TE insertion candidates. Third, conduct experimental validation of TE insertion candidates for their role in human brain evolution. Datasets and analytical outcomes are indicated with rounded rectangles; experimental procedures are indicated with rectangles; computational and statistical procedures are indicated with parallelograms. Dashed arrows indicate that after obtaining TE insertion profiles, researchers may use population genetic models and/or functional annotations to narrow down to a refined list of TE insertion candidates

TE-related human brain evolution studies are still in their infancy [33], but we present a potential future direction toward understanding the role of TEs in human brain evolution (Fig. 5). Given the lack of annotation of phenotypic impact of TE insertions, we suggest an unbiased search of evolutionarily important TEs using aDNA genomic data, complemented with brain functional data, such as RNA-seq from brain tissue and cell types. The complex and likely polygenic nature of brain-associated phenotypes calls for inclusion of both reference and polymorphic TE insertions. Integration of the findings from an unbiased search with comparative NHP analysis and GWAS results for cognitive traits and neurological diseases may prove instrumental in highlighting promising genetic candidates for experimental validations.

Despite the genomic focus of this review, we acknowledge that the environment is still a main contributor to the high functioning human brain and significantly complicates human brain evolution research [181]. Before investigating the natural selection, significant difficulty remains in selecting quantifiable phenotypes indicative of the higher functionality of the human brain [8]. Moreover, these phenotypes are likely to be polygenic traits for which genetics responds to selective pressure from the environment [107, 182]. To handle the complications from environmental influences, we need to combine knowledge from behavioral studies, anatomy, theoretical knowledge of complex systems, history, archaeology, and ancient genomes of animals and pathogens living alongside ancient humans [4, 51, 181].

## Supplementary Information

**Additional file 1: **previous_studies_with_ancient_data.xlsx. **Table S1**. Genome-wide aDNA data in addition to those curated in Marciniak and Perry *Nat Rev Genet* 2017. **Table S2**. Summary of genome-wide aDNA studies by year of publication and geographic region. The authors apologize for any accidental omissions of published ancient human or archaic hominin genomic data sets. These data are otherwise intended to be current as of December 2020. SNP, single-nucleotide polymorphism; WGS, whole-genome sequencing. * > 1× indicates an average coverage greater than 1-fold. ** > 5× indicates an average coverage greater than 5-fold. ‡Geographic region definitions: - Central Europe: Croatia, the Czech Republic, Hungary, Austria, Poland, Germany, Switzerland, Serbia, Slovakia. - Eastern Europe: Belarus, the Ukraine, Russia, Bulgaria, Montenegro, Caucasus, Romania, Moldova, Armenia. - Northern Europe: Estonia, Denmark, Sweden, Lithuania, Latvia, Ireland, United Kingdom, Norway, Netherlands, Belgium, France, Luxembourg, Finland, Iceland, Isle of Man. - Southern Europe: Spain, Portugal, Italy, Greece, Macedonia. - Asia: Nepal, China, India, Pakistan, Mongolia, Thailand, Cambodia, Vietnam, Laos, Malaysia, Indonesia, Philippines, Japan, Kazakhstan, Uzbekistan, Tajikistan, Turkmenistan, Afghanistan, Kyrgyzstan. - Middle East: Iran, Israel, Turkey, Jordan, Lebanon, Yemen. - North America: Canada, United States, Greenland, Central America, the Caribbean, Mexico, Bahamas, − South America: Brazil, Chile, Bolivia, Peru, Argentina, Venezuela. - Africa: Ethiopia, Egypt, Chad, Nigeria, Morocco. - Oceania: Vanuatu, Australia, Tonga, Solomon Islands, French Polynesia. §For subsets of samples without reported specific dates.

## Data Availability

Data sharing is not applicable to this article as no datasets were generated or analyzed during the current study.

## Abbreviations

1KGP: The 1000 Genomes Project
aDNA: Ancient human DNA
AF: Allele frequency
AMH: Anatomically modern human
APS: Adjusted proportion of singletons
CNV: Copy number variation
GWAS: Genome-wide association study
HAR: Human Accelerated Region
HGDP: The Human Genomes Diversity Project
iPSCs: Induced pluripotent stem cells
Ka/Ks: Ratio of non-synonymous and synonymous changes
KRAB: Kruppel-associated box
KZFP: KRAB zinc finger protein
LD: Linkage disequilibrium
lncRNA: Long non-coding RNA
LTR: Long terminal repeat
miRNA: microRNA
ncRNA: Non-coding RNA
NHP: Non-human primate
NPCs: Neuronal progenitor cells
PCA: Principal Component Analysis
rAAV: Recombinant adeno-associated virus
SD: Segmental duplication
SDS: Singleton Density Score
SGDP: The Simons Genome Diversity Project
SNP: Single-nucleotide polymorphism
snRNA-seq: Single-nuclei RNA sequencing
STR: Short tandem repeat
SV: Structural variation
TE: Transposable element
tSDS: Trait SDS
TSD: Target site duplication
TPRT: Target-primed reverse transcription
UDG: Uracil-DNA glycosylase
WGS: Whole-genome sequencing
XP-EHH: Cross Population Extended Haplotype Homozygosity
